# Madness Or—What? III.—The Kings of Spain

**Published:** 1894-09-15

**Authors:** 


### The Psychologist in History.

### MADNESS OR—WHAT?

### III.—The Kings of Spain.

Fond parents shed tears when the spoiled and petted son of their hopes marries, as they say, beneath him. Cautious and avaricious parents wish him to marry the heiress cousin, "keep the money in the family." Even the most complaisant and amiable parents wonder at his marrying a girl "with not a single taste in common with him." Hardly any father, one might say not a single mother, can see anything but misery in the choice the boy makes of a girl whose characteristics, physical and mental, are the very opposite of his own. But nature is wiser than the wisest parents, and guides the hearts of men and maidens to such union as will preserve a wholesome balance of qualities. Even so, one meets with families where one child seems to have all the strong points of both parents and another all the weaknesses; but on the whole the ordinary attraction of opposites results in a wholesome balance of qualities.

But, unfortunately, the higher the social sphere the narrower is the circle from which brides and bridegrooms may be chosen. Questions of policy decide the union even though there, more than elsewhere, it is the heir that is thought of rather than the bride. In Royal families the limit of choice is so small that after one or two generations it is almost impossible for a prince to avoid marrying his cousin one or two degrees removed. In the Royal family of Spain from the days of Ferdinand and Isabella till the extinction of the dynasty, within the space of eight generations whose lives extended over 350 years, we have a story of heredity which can hardly be equalled. Ferdinand and Isabella were themselves akin, both descended from the bold Henry of Trastamara, who usurped his brother's throne. But Isabella was also a descendant of that brother, Pedro, surnamed the Cruel, whom some have thought was mad, and certainly her mother, Isabella of Portugal, was insane. Both Ferdinand and his wife were sane enough—crafty, politic, judicious, cold-hearted, but sane. Wise as they were, however, they united in making their kingdom an absolute monarchy, and therefore forged a weapon which, as experience has too frequently proved, ends in ruining its possessors.

Of their four children only one, Juana, the second daughter, left any children, and certainly in her last years, if not, as seems likely, for many years before her death, Juana was insane. But her son was the great Emperor Charles V. It may be said that the epilepsy and fits of religious melancholy to which Charles V. was subject showed mental instability; but his illegitimate children, Don John and Margaret of Austria, both showed marked capacity and force of character, while the more dubious characteristics which marked him were reproduced in the children of his marriage with his cousin, Isabella of Portugal, in whose dynasty a history of insanity was to be found. His eldest son, Philip II., was gloomy, obstinate, and superstitious. His wife, who was also his cousin, was a princess of Portugal, and a grandchild of the insane Juana. Her mother was Catherine, Juana's youngest daughter, who in her youth had shared her mother's confinement in the convent prison of Tordesillas. The child of this couple was that Don Carlos, around whose name Schiller and Alfieri have woven a halo of romance which seems to have been little deserved. Don Carlos was ugly and deformed—one leg shorter than the other, a hump upon his back, and a malformation of the jaw, which interfered with his articulation. He was dull of intellect—he was five years old before he could even speak—and his tutors owned that he had no love for learning. He was equally deficient in manly exercises, and in temper he was wilful, capricious, and cruel. The diseased characteristics which marked his youth were aggravated by an accident he met with at the age of sixteen, which necessitated his skull being trephined, and nearly brought about his death. His father regarded him as mad, and when Carlos turned his malice against him, had him imprisoned. He died in prison at the age of twenty-three.

Philip was four times married. By the last marriage—with his niece, daughter of his sister Mary—he left a son, who inherited the throne. This son, Philip III., was weak and indolent, and left the government of his country to his favourites. His successor, Philip IV., though not destitute of literary talent, was a poor ruler, being both lazy and immoral. To him succeeded his younger son, Charles II. In this monarch all the weaknesses of the race culminated. From his birth he was sickly; indeed, until he was ten years of age he never set his foot on the ground. He could acquire no education—even the history of the country over which he was supposed to rule was a blank to him. The affairs of his kingdom could not command his attention; but at least, in early life, he showed some liking for athletic sports. "He enjoyed," says Macaulay, "with the delight of a true Spaniard, two delightful spectacles, a horse with its bowels gored out and a Jew writhing in the fire." After the death of his first wife, whom he loved after his fashion, a gloomy religious mania took possession of him. He fasted, he whipped himself, he walked in processions. Every year he grew more gloomy. Sometimes he shut himself up in an inner room of the palace, sometimes he wandered alone from dawn to dusk. The family malformation of the jaw, a legacy from the great Emperor, was so marked in him as to make mastication impossible, and, as a consequence, his digestion gave way. He died, childless, in 1700, leaving a disputed succession to embroil all Europe.

The hereditary weaknesses of the Spanish Royal Family were undoubtedly aggravated by the frequent intermarriages of its members. Every bride, cousin, or niece of the same blood brought back some portion of the family qualities to strengthen those in the direct line. The strongest strain would fail under this. Qualities good in themselves become aggravated into their corresponding vices by such unions; the true balance of nature is shaken. And in this family there was from the beginning a neurotic taint, which showed itself in various ways, sometimes as epilepsy, and again as melancholia, madness, or imbecility. How much these weaknesses were increased by the possession of absolute power is an interesting and difficult question, which we hope to illustrate by future examples.

